# Increasing the Focus on Children's Complex and Integrated Care Needs: A Position Paper of the European Academy of Pediatrics

**DOI:** 10.3389/fped.2021.758415

**Published:** 2021-12-01

**Authors:** Maria Brenner, Josephine Greene, Carmel Doyle, Berthold Koletzko, Stefano del Torso, Ivan Bambir, Ann De Guchtenaere, Theofilos Polychronakis, Laura Reali, Adamos A. Hadjipanayis

**Affiliations:** ^1^European Academy of Paediatrics (EAP), Brussels, Belgium; ^2^School of Nursing & Midwifery, Trinity College Dublin, Dublin, Ireland; ^3^Dr. Von Hauner Children's Hospital, Ludwig Maximilian University of Munich, Munich, Germany; ^4^ChildCare WorldWide, Padova, Italy; ^5^Department of Pediatrics, University Hospital Centre Zagreb, Zagreb, Croatia; ^6^Department of Pediatrics, Ghent University Hospital, Ghent, Belgium; ^7^Cambridge University Hospitals NHS Foundation Trust, Cambridge, United Kingdom; ^8^Studio Pediatrico, Rome, Italy; ^9^Paediatric Department, Larnaca General Hospital, Larnaca, Cyprus; ^10^School of Medicine, European University Cyprus, Nicosia, Cyprus

**Keywords:** care coordination, child, community, complex care, family, integrated care

## Abstract

There is wide variation in terminology used to refer to children living with complex needs, across clinical, research and policy settings. It is important to seek to reconcile this variation to support the effective development of programmes of care for this group of children and their families. The European Academy of Pediatrics (EAP) established a multidisciplinary Working Group on Complex Care and the initial work of this group examined how complex care is defined in the literature. A scoping review was conducted which yielded 87 papers with multiple terms found that refer to children living with complex needs. We found that elements of integrated care, an essential component of care delivery to these children, were repeatedly referred to, though it was never specifically incorporated into a term to describe complex care needs. This is essential for practice and policy, to continuously assert the need for integrated care where a complex care need exists. We propose the use of the term Complex and Integrated Care Needs as a suitable term to refer to children with varying levels of complexity who require continuity of care across a variety of health and social care settings.

## Introduction

A number of variations of the term complex care are used in practice, which in its broadest sense refers to children living with “multidimensional health and social care needs in the presence of a recognized medical condition or where there is no unifying diagnosis” (1). There has been a strong emphasis in North America in particular to move from broader definitions of complex care to more focused terminology. This has been identified as favorable in terms of the organization of hospital-led care for children with more severe complexities. However, it has been questioned if this change in name would lead to meaningful changes in care outcomes, and what a change in terminology would mean for children with less severe complex needs who are cared for predominantly in the community (2).

From a European perspective there is wide variation in terminology used across clinical, research and policy settings. It is important to seek to reconcile this variation and to address the absence of agreed terminology. This is important: to support the effective development of programmes of care for this group of children and their families; for connectivity across health and social care systems and services; for interprofessional communication, education, and research; and for meaningful policy development. It is also important that any definition agreed is not used to negatively impact on resources available for any other group of children and their families. To examine this and other developing issues in the care of children with complex needs the European Academy of Pediatrics (EAP) established a multidisciplinary Working Group on Complex Care in Spring 2021. The overall aim of the work of this group is to support multidisciplinary practice, education and research on children's complex care in Europe. The initial work of this group was to examine how complex care is defined in the literature. In order to begin to understand the various terms used to portray complex care, we conducted a scoping review to establish what terms have already been used in the literature. Here we report our key considerations and conclusions on this issue.

## Methods

A systematic search of literature was initially conducted to identify all of the current published variations in definitions of complex care in children. To search effectively and strategically, the review question was broken down into constituent parts and the literature was searched with Boolean operators using the following criteria: population search terms (child or children or youth or young person or young people or family or families or parent or adolescent or p(a)ediatric or p(a)ediatrics); and issue/intervention search terms: (complex needs or complex care or complex medical care or medical complexity or chronic illness or chronic disease or chronically complex or chronic health condition or chronic* or complex chronically ill disability or disab* or healthcare or serious illness or special healthcare needs or special health care needs or life-limiting condition).

It was required that the term complex must be contained in either the article title or abstract. Finally, in addition to matching search terms and keywords, the documents for review had to meet the following inclusion criteria: must be published between January 2016 and April 2021 (to reflect current terminology in use) and the articles had to be published in a peer reviewed journal. Studies not available in English language were excluded.

**Figure 1:**
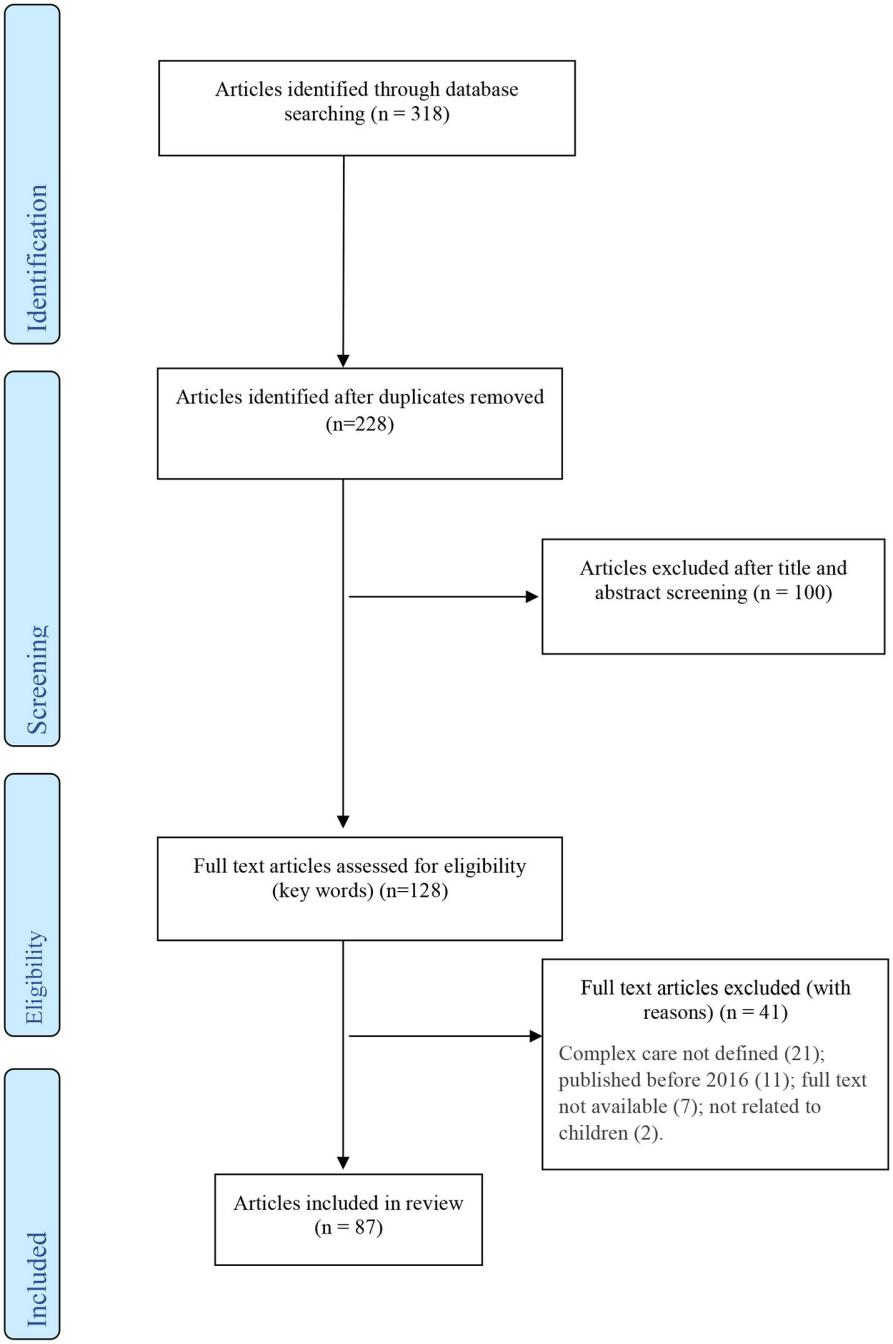
Data search and selection process.

In total, 318 documents were identified for inclusion and were imported to Mendeley reference manager (Figure 1). After removal of duplications, 228 studies were imported to Covidence screening and data extraction tool. An agreed screening protocol was devised and agreed among the research team. MB and JG reviewed the titles and abstracts for compliance with the predefined inclusion criteria. One hundred and twenty-eight studies were deemed eligible for full-text screening. JG screened the titles, abstract and full text of the selected articles. After the full text review, an additional 41 studies were excluded based on the pre-defined criteria, resulting in 87 articles for data extraction. The primary reason for excluding articles at this stage was that complex care was not explicitly defined (*n* = 21). Other reasons included that the article was published before 2016 (*n* = 11); the full text of the article was not available (*n* = 7); or the paper was not related to children (*n* = 2).

### Data Extraction

A data charting form was developed to capture the various terms used to portray complex care in the literature. This chart consisted of the following items: journal citation; country; aim of article; definition of complex care; limitations; and recommendations. The data charting forms were independently examined by two reviewers (JG and MB) to ensure the data gathered could address the aim of this review.

## Findings

The three main terms that repeatedly emerged in the data reviewed referred to children with medical complexity (*n* = 46), children with complex care needs (*n* = 13), and children with (special) healthcare needs (*n* = 17). The terms complex care needs and special healthcare needs most commonly referred to children with both medical and social care needs, while the term medical complexity was often referred to as a subset of children of these groups and was most commonly used to indicate a group of children with a very high level of specific medical complexities. A very small number of articles used the term complex care when referring to the needs of children living with life-limiting conditions, children living with a chronic critical illness or used the term in conjunction with less commonly used terms (for example catastrophic medical complexity) (*n* = 7). Table 1 presents the full list of terms found in the articles reviewed and the Supplementary File includes detail of the associated references for each term. The term integrated care needs was not explicitly used in the articles reviewed to describe the needs of children with complex care needs.

**Table 1:** Terms used in the literature to refer to children with complex care needs.

**Terms identified in the literature**	**Number of articles identified**
Children with Medical Complexity (CMC)	46
Pediatric Medical Complexity	
Complex care needs (CCN)	13
Children's complex care needs	
Children with complex needs	
Multiple complex needs (MCN)	
Complex healthcare needs	17
Complex health care needs	
Special healthcare needs (SHCN)	
Children with special health care needs (CSHCN)	
Children and Youth with special health care needs (CYSHCN)	
Complex chronic conditions	7
Complex Medical Conditions	
Catastrophic medical complexity	
Children with life-limiting conditions	
Chronic Critical Illness (CCI)	
Pediatric Chronic Critical Illness (PCCI)	
Multiple terms	4
	Total: 87

## Discussion

The findings of the review were presented and discussed at length within the Working Group on Complex Care, with the aim of identifying an optimum term that could be meaningfully used across the variety of cultures and healthcare settings in Europe. Each term relating to complex care identified in the literature was examined in detail, taking into account clinical and academic observations across acute and community care across Europe. This included discussion on the use and placement of the word complex in the terms, and the meaning and associations of the other words used in each term found.

While there were a large variety of terms, it was noted that the concept of integrated care was mentioned in association with complex care in the majority of the articles reviewed, though never explicitly used in a term to describe children with complex needs. For example, poorly developed integrated care services have been identified as a barrier to comprehensive care delivery for children with complex needs (3). An integrated care approach was identified as necessary to support a person-centered approach to the care of children with complex care needs (4, 5), while cross-sector, integrated and assertive care delivery was identified as necessary for safeguarding the well-being and development of children with complex care needs (6). Principles of integrated care were also frequently mentioned in the literature reviewed (7–11).

Integrated care is an essential component of care delivery to children with complex needs and the literature is replete with examples of important developments to support connected and integrated care, with an emphasis on the need for flexibility in service delivery, and pathways of care that are individualized for each child and their family. Models, frameworks and principles of care have been developed to support integration of care (3, 12) however, despite an increase in initiatives in this area it is insufficient to meet the needs of these children. There is continuous evidence internationally that many children with complex needs have ongoing issues with access to appropriate and continuous care in the home, access to respite care services, and challenges in communication within and across services (13–16). There is also continuous reference to the need for a care coordinator in the community to support the integration of their care needs (9, 17, 18).

There is a clear link between having a complex need and requiring integrated care, however, the concept of integrated care was never specifically incorporated into a term to describe complex care needs. This is unusual given the fact that the core principle of integrated care is to bring together key aspects of care to avoid fragmentation in care delivery. We would suggest that there is a need to combine the terms so that they are continuously identified as synonymous and thereby continuously highlight that a complex care need is an integrated care need.

The absence of this combination in any formal manner in the literature may be explained by the exponential rise in varying terminologies in the area of integrated care. Over the last 20 years numerous taxonomies or typologies of integrated care emerged referring to different types, forms or models of integrated care (19), to the extent that there has been development and validation of two specific search filters for rapid and effective retrieval of integrated care evidence (20). The review of the literature identifies the potential for deliberations on terminology on complex care to follow a similar complicated path of typologies leaving it potentially challenging over time to use in a practically useful way. While the debates on terminology continue there remains a need for a clear term that can be easily understood and applied in health and social care settings across a wide variety of cultural and geographically diverse regions. This is essential for practice and policy to continuously assert the need for integrated care where a complex care need exists. We would therefore propose the use of the term Complex and Integrated Care Needs as a suitable term to refer to children with varying levels of complexity who required continuity of care across a variety of health and social care settings.

## Conclusion

The aim of this paper was to identify a meaningful term for the care of children with complex needs that could be used by children and their families, health and social care workers, academics and policy makers, across a wide cultural and geographically disperse region. The literature highlighted numerous terms that are used to describe children with complex needs, with a dominance of three main terms: children with medical complexity, children with complex care needs, and children with (special) healthcare needs. While the Working Group agreed that the broader term complex care needs is the most accommodating of these three main terms (as it refers to both health and social care needs), they concluded that this term does not reflect the fact that a complex care need is an integrated care need. The Working Group concluded that the term complex care alone does not reflect the importance of a flexible and dynamic service that can provide individualized care for each child. The Working Group on Complex Care therefore proposes that the term **Complex and Integrated Care Needs** is a suitable term to refer to children with varying levels of complexity who require continuity of care across a variety of health and social care settings.

## Data Availability Statement

The original contributions presented in the study are included in the article/Supplementary Material, further inquiries can be directed to the corresponding author/s.

## Author Contributions

MB and JG completed the search and analysis of the literature. All authors conceptualized the paper, contributed to the article development and approved the submitted version.

## Conflict of Interest

The authors declare that the research was conducted in the absence of any commercial or financial relationships that could be construed as a potential conflict of interest.

## Publisher's Note

All claims expressed in this article are solely those of the authors and do not necessarily represent those of their affiliated organizations, or those of the publisher, the editors and the reviewers. Any product that may be evaluated in this article, or claim that may be made by its manufacturer, is not guaranteed or endorsed by the publisher.
