# Supplementary material for: Increasing the Focus on Children's Complex and Integrated Care Needs: A Position Paper of the European Academy of Pediatrics
Source: Front Pediatr. 2021 Dec 1;9:758415. doi: 10.3389/fped.2021.758415 (PMC8671931; doi:10.3389/fped.2021.758415)
Supplement: Supplementary file 1 [file Data_Sheet_1.PDF]

EAP Position Paper

Table 1

**Terms identified in the literature**	**Number of articles identified**
Children with Medical Complexity (CMC)[1,3,8,11,16,17,18,20,24,27,29,30,31,32,36,37,38,39,40,41,42,43,44,45,49,50,53,54,55,56,57,59,60,61,62,66,67,69,70,72,73,74,78,81,82]<br>Pediatric Medical Complexity [87]	46
Complex care needs (CCN) [13,46,47]<br>Children's complex care needs [14,19]<br>Children with complex needs [23,25,79,86]<br>Multiple complex needs (MCN)[22,34,75,76,]	13
Complex healthcare needs [15,63]<br>Complex health needs [84,85]<br>Special healthcare needs (SHCN) [77,83]<br>Children with special health care needs (CSHCN) [2,7,10,33,48,52,64,65]<br>Children and Youth with special health care needs (CYSHCN) [5,6,80]	17
Complex chronic conditions [28]<br>Complex Medical Conditions [35]<br>Catastrophic medical complexity [58]<br>Children with life-limiting conditions [26,51]<br>Chronic Critical Illness (CCI) [68,]<br>Pediatric Chronic Critical Illness (PCCI) [71]	7
Multiple Terms [4,9,12,21,]	4
	Total: 87

Supplementary File

Reference List associated with Table 1

1. Abebe, E. *et al.* (2020) 'What do family caregivers do when managing medications for their children with medical complexity?', *Applied ergonomics*. England, 87, p. 103108. doi: 10.1016/j.apergo.2020.103108.
2. Aboneh, E. A. and Chui, M. A. (2017) 'Care coordination, medical complexity, and unmet need for prescription medications among children with special health care needs', *Research in Social and Administrative Pharmacy*. Elsevier Inc., 13(3), pp. 524–529. doi: 10.1016/j.sapharm.2016.05.043.
3. Adams, S. *et al.* (2017) 'Care maps for children with medical complexity', *Dev Med Child Neurol*. Blackwell Publishing Ltd, 59(12), pp. 1299–1306. doi: 10.1111/dmcn.13576.
4. Allen, J. *et al.* (2020) 'Severe Neurological Impairment: A delphi consensus-based definition', *European Journal of Paediatric Neurology*. W.B. Saunders Ltd, 29, pp. 81–86. doi: 10.1016/j.ejpn.2020.09.001.

5. Anderson, B. *et al.* (2017) 'The eye of the beholder: A discussion of value and quality from the perspective of families of children and youth with special health care needs', *Pediatrics*. American Academy of Pediatrics, 139, pp. S99–S108. doi: 10.1542/peds.2016-2786D.
6. Bachman, S. S., Comeau, M. and Long, T. F. (2017) 'Statement of the problem: Health reform, value-based purchasing, alternative payment strategies, and children and youth with special health care needs', *Pediatrics*. American Academy of Pediatrics, 139, pp. S89–S98. doi: 10.1542/peds.2016-2786C.
7. Balistreri, K. S. (2019) 'Food insufficiency and children with special healthcare needs.', *Public health*. Elsevier B.V., 167, pp. 55–61. doi: 10.1016/j.puhe.2018.11.011.
8. Barnert, E. S. *et al.* (2019) 'Key Population Health Outcomes for Children with Medical Complexity: A Systematic Review.', *Maternal and child health journal*. United States: Springer New York LLC, 23(9), pp. 1167–1176. doi: 10.1007/s10995-019-02752-1.
9. Bradshaw, S. *et al.* (2019) 'Improving health, wellbeing and parenting skills in parents of children with special health care needs and medical complexity - a scoping review.', *BMC pediatrics*, 19(1), p. 301. doi: 10.1186/s12887-019-1648-7.
10. Braganza, S. F. *et al.* (2020) 'CORNET Card Study #1: Do You See What I See? Provider Confidence in Caring for Children With Special Health Care Needs.', *Academic pediatrics*. United States, 20(2), pp. 250–257. doi: 10.1016/j.acap.2019.10.005.
11. Breneol, S. *et al.* (2017) 'Strategies to support transitions from hospital to home for children with medical complexity: A scoping review', *International Journal of Nursing Studies*. Elsevier Ltd, pp. 91–104. doi: 10.1016/j.ijnurstu.2017.04.011.
12. Breneol, S. *et al.* (2019) 'Respite care for children and youth with complex care needs and their families: a scoping review protocol.', *JBI database of systematic reviews and implementation reports*. Australia: NLM (Medline), 17(7), pp. 1297–1304. doi: 10.11124/JBISRIR-2017-003998.
13. Brenner, M., Kidston, C., *et al.* (2018) 'Children's complex care needs: a systematic concept analysis of multidisciplinary language', *European Journal of Pediatrics*. Springer Verlag, 177(11), pp. 1641–1652. doi: 10.1007/s00431-018-3216-9.
14. Brenner, M., O'Shea, M. P., *et al.* (2018) 'Management and integration of care for children living with complex care needs at the acute-community interface in Europe.', *The Lancet. Child & adolescent health*. England, 2(11), pp. 822–831. doi: 10.1016/S2352-4642(18)30272-4.
15. Brenner, M. *et al.* (2021) 'Enhancing care of children with complex healthcare needs: an improvement project in a community health organisation in Ireland.', *BMJ open quality*, 10(1). doi: 10.1136/bmjoq-2020-001025.
16. Cady, R. *et al.* (2020) 'Care coordination for children with medical complexity.', *The Nurse practitioner*. United States: NLM (Medline), 45(6), pp. 11–17. doi: 10.1097/01.NPR.0000666172.10978.4f.
17. Cohen, E. *et al.* (2018) 'Status Complexicus? The Emergence of Pediatric Complex Care.', *Pediatrics*. United States, 141(Suppl 3), pp. S202–S211. doi: 10.1542/peds.2017-1284E.
18. Coller, R. J. *et al.* (2019) 'Variation in Hospitalization Rates Following Emergency Department Visits in Children with Medical Complexity.', *The Journal of pediatrics*. United States, 214, pp. 113-120.e1. doi: 10.1016/j.jpeds.2019.07.034.

19. Curran, J. A., Breneol, S. and Vine, J. (2020) 'Improving transitions in care for children with complex and medically fragile needs: a mixed methods study.', *BMC pediatrics*. BioMed Central Ltd., 20(1), p. 219. doi: 10.1186/s12887-020-02117-6.
20. D'Aprano, A. *et al.* (2020) 'Important components of a programme for children with medical complexity: An Australian perspective.', *Child: care, health and development*. England, 46(1), pp. 90–103. doi: 10.1111/cch.12721.
21. Diffin, J. *et al.* (2019) 'The usefulness and acceptability of a personal health record to children and young people living with a complex health condition: A realist review of the literature.', *Child: care, health and development*, 45(3), pp. 313–332. doi: 10.1111/cch.12652.
22. Van Dongen, T., Sabbe, B. and Glazemakers, I. (2020) 'Collaboration for children with complex needs: What adolescents, parents, and practitioners tell us.', *Journal of child health care : for professionals working with children in the hospital and community*. England: SAGE Publications Inc., 24(1), pp. 19–32. doi: 10.1177/1367493518823906.
23. Doucet, S. *et al.* (2020) 'Programmes to support transitions in care for children and youth with complex care needs and their families: a scoping review protocol.', *BMJ open*, 10(6), p. e033978. doi: 10.1136/bmjopen-2019-033978.
24. Edelstein, H. *et al.* (2017) 'Children with medical complexity: a scoping review of interventions to support caregiver stress', *Child: Care, Health and Development*. Blackwell Publishing Ltd, pp. 323–333. doi: 10.1111/cch.12430.
25. Fraser, L. *et al.* (2020) 'Long-term quality of life in children with complex needs undergoing cochlear implantation.', *International journal of pediatric otorhinolaryngology*. Ireland: Elsevier Ireland Ltd, 136, p. 110223. doi: 10.1016/j.ijporl.2020.110223.
26. Fraser, L. K. *et al.* (2020) 'Estimating the current and future prevalence of life-limiting conditions in children in England.', *Palliative medicine*. England: SAGE Publications Ltd, p. 269216320975308. doi: 10.1177/0269216320975308.
27. Fratantoni, K. *et al.* (2019) 'The Pediatric Home Health Care Process: Perspectives of Prescribers, Providers, and Recipients.', *Pediatrics*. United States: American Academy of Pediatrics, 144(3). doi: 10.1542/peds.2019-0897.
28. Friedel, M. *et al.* (2019) 'Access to paediatric palliative care in children and adolescents with complex chronic conditions: a retrospective hospital-based study in Brussels, Belgium.', *BMJ paediatrics open*, 3(1), p. e000547. doi: 10.1136/bmjpo-2019-000547.
29. Friedman, S. L. *et al.* (2016) 'Out-of-home placement for children and adolescents with disabilities-Addendum: Care options for Children and adolescents with disabilities and medical complexity', *Pediatrics*. American Academy of Pediatrics, 138(6). doi: 10.1542/peds.2016-3216.
30. Gallo, M. *et al.* (2021) 'The child with medical complexity', *Italian Journal of Pediatrics*. BioMed Central Ltd. doi: 10.1186/s13052-020-00935-z.
31. Gay, J. C. *et al.* (2016) 'Home health nursing care and hospital use for medically complex children', *Pediatrics*. American Academy of Pediatrics, 138(5). doi: 10.1542/peds.2016-0530.
32. Glader, L., Plews-Ogan, J. and Agrawal, R. (2016) 'Children with medical complexity: creating a framework for care based on the International Classification of Functioning, Disability and Health', *Developmental Medicine and Child Neurology*.

Blackwell Publishing Ltd, pp. 1116–1123. doi: 10.1111/dmcn.13201.
33. Glassman, P. (2017) ‘Interventions Focusing on Children with Special Health Care Needs’, *Dental Clinics of North America*. W.B. Saunders, pp. 565–576. doi: 10.1016/j.cden.2017.02.007.
34. Heggestad, T. *et al.* (2020) ‘Complex care pathways for children with multiple referrals demonstrated in a retrospective population-based study.’, *Acta paediatrica (Oslo, Norway : 1992)*. Norway: Blackwell Publishing Ltd, 109(12), pp. 2641–2647. doi: 10.1111/apa.15250.
35. Hessels, A. J. *et al.* (2020) ‘Patient Safety Culture Survey in Pediatric Complex Care Settings: A Factor Analysis.’, *Journal of patient safety*, 16(3), pp. 223–231. doi: 10.1097/PTS.0000000000000279.
36. Huth, K. *et al.* (2018a) ‘Approach to Clinical Assessment of Children With Medical Complexity.’, *MedEdPORTAL : the journal of teaching and learning resources*, 14, p. 10765. doi: 10.15766/mep_2374-8265.10765.
37. Huth, K. *et al.* (2018b) ‘Understanding the Needs of Children With Medical Complexity.’, *MedEdPORTAL : the journal of teaching and learning resources*. NLM (Medline), 14, p. 10709. doi: 10.15766/mep_2374-8265.10709.
38. Huth, K. *et al.* (2020) ‘Evaluating Curricular Modules in the Care of Children With Medical Complexity: A Mixed-Methods Randomized Controlled Trial.’, *Academic pediatrics*. United States, 20(2), pp. 282–289. doi: 10.1016/j.acap.2019.09.002.
39. Huth, K., Newman, L. and Glader, L. (2020) ‘Core Curricular Priorities in the Care of Children With Medical Complexity: A North American Modified Delphi Study.’, *Academic pediatrics*. United States, 20(4), pp. 558–564. doi: 10.1016/j.acap.2020.01.014.
40. Lawrence, P. R., Feinberg, I. and Spratling, R. (2021) ‘The relationship of parental health literacy to health outcomes of children with medical complexity.’, *Journal of pediatric nursing*. United States, 60, pp. 65–70. doi: 10.1016/j.pedn.2021.02.014.
41. Leary, J. C. *et al.* (2019) ‘Developing Prediction Models for 30-Day Unplanned Readmission Among Children With Medical Complexity.’, *Hospital pediatrics*, 9(3), pp. 201–208. doi: 10.1542/hpeds.2018-0174.
42. Leary, J. C. *et al.* (2020) ‘Parent Perspectives During Hospital Readmissions for Children With Medical Complexity: A Qualitative Study.’, *Hospital pediatrics*, 10(3), pp. 222–229. doi: 10.1542/hpeds.2019-0185.
43. Lin, J. L., Cohen, E. and Sanders, L. M. (2018) ‘Shared Decision Making among Children with Medical Complexity: Results from a Population-Based Survey.’, *The Journal of pediatrics*, 192, pp. 216–222. doi: 10.1016/j.jpeds.2017.09.001.
44. Lindstrom, K., Cady, R. and Bushaw, A. (2020) ‘Family-centered care for children with medical complexity: A goal-planning initiative.’, *The Nurse practitioner*. United States, 45(8), pp. 49–55. doi: 10.1097/01.NPR.0000681796.57869.a0.
45. Lord, S. *et al.* (2020) ‘Assessment of Bereaved Caregiver Experiences of Advance Care Planning for Children with Medical Complexity’, *JAMA Network Open*. American Medical Association, 3(7). doi: 10.1001/jamanetworkopen.2020.10337.
46. Luck, K. E., Doucet, S. and Luke, A. (2020) ‘The Development of a Logic Model to Guide the Planning and Evaluation of a Navigation Center for Children and Youth with Complex Care Needs’, *Child and Youth Services*. Routledge, 41(4), pp. 327–341. doi: 10.1080/0145935X.2019.1684192.
47. Luke, A., Luck, K. E. and Doucet, S. (2020) ‘Experiences of Caregivers as Clients of a

Patient Navigation Program for Children and Youth with Complex Care Needs: A Qualitative Descriptive Study.', *International journal of integrated care*, 20(4), p. 10. doi: 10.5334/ijic.5451.
48. Matiz, L. A., Robbins-Milne, L. and Rausch, J. A. (2019) 'EMR Adaptations to Support the Identification and Risk Stratification of Children with Special Health Care Needs in the Medical Home', *Maternal and Child Health Journal*. Springer New York LLC, 23(7), pp. 919–924. doi: 10.1007/s10995-018-02718-9.
49. Ming, D. Y. *et al.* (2019) 'Mobile Complex Care Plans to Enhance Parental Engagement for Children With Medical Complexity', *Clinical Pediatrics*. SAGE Publications Inc., 58(1), pp. 34–41. doi: 10.1177/0009922818805241.
50. Ming, D. Y. *et al.* (2020) 'The Intersection of Complex Care and Hospital Medicine: Opportunities to Advance Health for Chronically Ill Populations.', *Hospital pediatrics*. United States, 10(8), pp. 715–718. doi: 10.1542/hpeds.2020-0079.
51. Mitchell, S. *et al.* (2021) 'Experiences of general practice of children with complex and palliative care needs and their families: a qualitative study.', *BMJ open*, 11(1), p. e041476. doi: 10.1136/bmjopen-2020-041476.
52. Moeenuddin, Z. *et al.* (2019) 'The Influence of Care Coordination on Patients With Special Health Care Needs in a Pediatric Residency Continuity Clinic.', *Global pediatric health*, 6, p. 2333794X19848677. doi: 10.1177/2333794X19848677.
53. Mooney-Doyle, K. and Lindley, L. C. (2020) 'Family and Child Characteristics Associated With Caregiver Challenges for Medically Complex Children.', *Family & community health*. United States: Lippincott Williams and Wilkins, 43(1), pp. 74–81. doi: 10.1097/FCH.0000000000000245.
54. Morse, B. L. *et al.* (2020) 'Leveraging Parent Pain Perspectives to Improve Pain Practices for Children with Medical Complexity.', *Pain management nursing : official journal of the American Society of Pain Management Nurses*. United States. doi: 10.1016/j.pmn.2020.11.011.
55. Murphy, S. and Ehritz, C. (2021) 'Clinical Nurse Specialist Practice Strategies for Children With Medical Complexity.', *Clinical nurse specialist CNS*. United States, 35(1), pp. 38–43. doi: 10.1097/NUR.0000000000000567.
56. Nackers, A. *et al.* (2019) 'Encounters From Device Complications Among Children With Medical Complexity.', *Hospital pediatrics*, 9(1), pp. 6–15. doi: 10.1542/hpeds.2018-0103.
57. Nageswaran, S. *et al.* (2020) 'Randomized controlled trial evaluating a collaborative model of care for transitioning children with medical complexity from hospital to home healthcare: Study protocol.', *Contemporary clinical trials communications*, 20, p. 100652. doi: 10.1016/j.conctc.2020.100652.
58. Nasir, A. *et al.* (2018) 'Complexity in pediatric primary care.', *Primary health care research & development*. England: Cambridge University Press, pp. 1–7. doi: 10.1017/S146342361800035X.
59. Notario, P. M. *et al.* (2019) 'Home-Based Telemedicine for Children with Medical Complexity.', *Telemedicine journal and e-health : the official journal of the American Telemedicine Association*. Mary Ann Liebert Inc., 25(11), pp. 1123–1132. doi: 10.1089/tmj.2018.0186.
60. Onofri, A. *et al.* (2021) 'Telemedicine in children with medical complexity on home ventilation during the COVID-19 pandemic.', *Pediatric pulmonology*. doi: 10.1002/ppul.25289.

61. Orkin, J. *et al.* (2020) 'Toward an understanding of advance care planning in children with medical complexity', *Pediatrics*. American Academy of Pediatrics, 145(3). doi: 10.1542/peds.2019-2241.
62. Page, B. F. *et al.* (2020) 'The challenges of caring for children who require complex medical care at home: "The go between for everyone is the parent and as the parent that's an awful lot of responsibility".', *Health expectations : an international journal of public participation in health care and health policy*, 23(5), pp. 1144–1154. doi: 10.1111/hex.13092.
63. Peters, V. J. T. *et al.* (2020) 'Providing person-centered care for patients with complex healthcare needs: A qualitative study.', *PloS one*, 15(11), p. e0242418. doi: 10.1371/journal.pone.0242418.
64. Petitgout, J. M. (2018) 'The Financial Impact of a Hospital-Based Care Coordination Program for Children With Special Health Care Needs.', *Journal of pediatric health care : official publication of National Association of Pediatric Nurse Associates & Practitioners*. United States, 32(1), pp. 3–9. doi: 10.1016/j.pedhc.2017.06.003.
65. Precce, M. L. *et al.* (2020) 'Educational demands of family members of children with special health care needs in the transition from hospital to home.', *Revista brasileira de enfermagem*. Brazil, 73 Suppl 4, p. e20190156. doi: 10.1590/0034-7167-2019-0156.
66. Rennick, J. E. *et al.* (2019) 'Exploring the experiences of parent caregivers of children with chronic medical complexity during pediatric intensive care unit hospitalization: an interpretive descriptive study.', *BMC pediatrics*, 19(1), p. 272. doi: 10.1186/s12887-019-1634-0.
67. Rogers, J. *et al.* (2021) 'Children with medical complexity: A concept analysis.', *Nursing forum*. United States. doi: 10.1111/nuf.12559.
68. Rogozinski, L. *et al.* (2019) 'Point Prevalence of Children Hospitalized With Chronic Critical Illness in the General Inpatient Units', *Hosp Pediatr.* American Academy of Pediatrics (AAP), 9(7), pp. 545–549. doi: 10.1542/hpeds.2018-0208.
69. Ross, M. H. *et al.* (2020) 'Telemedicine Video Visits for Children with Medical Complexity in a Structured Clinical Complex Care Program.', *Global pediatric health*, 7, p. 2333794X20952196. doi: 10.1177/2333794X20952196.
70. Sadof, M. *et al.* (2019) 'A Step-by-Step Guide to Building a Complex Care Coordination Program in a Small Setting.', *Clinical pediatrics*. United States, 58(8), pp. 897–902. doi: 10.1177/0009922819849057.
71. Shapiro, M. C. *et al.* (2017) 'Defining Pediatric Chronic Critical Illness for Clinical Care, Research, and Policy', *Hospital pediatrics*, pp. 236–244. doi: 10.1542/hpeds.2016-0107.
72. Shimmura, K. and Tadaka, E. (2018) 'Development of an interprofessional collaboration competency scale for children with medical complexity.', *BMJ open*, 8(6), p. e019415. doi: 10.1136/bmjopen-2017-019415.
73. Sobush, K. T. (2019) 'Principles of Managing Children with Medical Complexity and a New Delivery Model.', *Missouri medicine*, 116(2), pp. 134–139.
74. Statile, A. M. *et al.* (2016) 'Improving discharge efficiency in medically complex pediatric patients', *Pediatrics*. American Academy of Pediatrics, 138(2). doi: 10.1542/peds.2015-3832.
75. Van den Steene, H., van West, D. and Glazemakers, I. (2018) 'A multi-perspective exploration of the service needs of adolescent girls with multiple and complex

needs', *Children and Youth Services Review*. Elsevier Ltd, 90, pp. 28–37. doi: 10.1016/j.childyouth.2018.05.010.
76. Van den Steene, H., van West, D. and Glazemakers, I. (2019) 'Towards a definition of multiple and complex needs in children and youth: Delphi study in Flanders and international survey.', *Scandinavian journal of child and adolescent psychiatry and psychology*. Exeley, Inc., 7, pp. 60–67. doi: 10.21307/sjcapp-2019-009.
77. Taddei, M. *et al.* (2016) 'Orthodontic treatment of a particular subgroup of children with special health care needs, children with craniofacial anomalies: An analysis of treatment length and clinical outcome', *Angle Orthodontist*. Allen Press Inc., 86(1), pp. 115–120. doi: 10.2319/122014.1.
78. Thomson, J. *et al.* (2016) 'Financial and social hardships in families of children with medical complexity', *Journal of Pediatrics*. Mosby Inc., 172, pp. 187-193.e1. doi: 10.1016/j.jpeds.2016.01.049.
79. Tobin, L. M. and Ebbels, S. H. (2019) 'Effectiveness of intervention with visual templates targeting tense and plural agreement in copula and auxiliary structures in school-aged children with complex needs: a pilot study.', *Clinical linguistics & phonetics*. England, 33(1–2), pp. 175–190. doi: 10.1080/02699206.2018.1501608.
80. Ufer, L. G. *et al.* (2018) 'Care Coordination: Empowering Families, a Promising Practice to Facilitate Medical Home Use Among Children and Youth with Special Health Care Needs.', *Maternal and child health journal*, 22(5), pp. 648–659. doi: 10.1007/s10995-018-2477-2.
81. Walter, A. W., Ellis, R. P. and Yuan, Y. (2019) 'Health care utilization and spending among privately insured children with medical complexity.', *Journal of child health care : for professionals working with children in the hospital and community*. England: SAGE Publications Inc., 23(2), pp. 213–231. doi: 10.1177/1367493518785778.
82. Ware, E. J. *et al.* (2020) 'Practical Needs in the Home Care of Latino Children With Medical Complexity.', *Home healthcare now*. United States: Lippincott Williams and Wilkins, 38(4), pp. 202–208. doi: 10.1097/NHH.0000000000000854.
83. Weeks, M. *et al.* (2018) 'Resident and Staff Satisfaction of Pediatric Graduate Medical Education Training on Transition to Adult Care of Medically Complex Patients.', *Military medicine*. England, 183(11–12), pp. e676–e679. doi: 10.1093/milmed/usy057.
84. Whiting, M. (2019) 'Caring for children - "24-7": The experience of WellChild Nurses and the families for whom they are providing care and support.', *Journal of child health care : for professionals working with children in the hospital and community*. England, 23(1), pp. 35–44. doi: 10.1177/1367493518777149.
85. Whiting, M. *et al.* (2019) 'Enhancing resilience and self-efficacy in the parents of children with disabilities and complex health needs.', *Primary health care research & development*, 20, p. e33. doi: 10.1017/S1463423619000112.
86. Wittmeier, K. D. M. *et al.* (2016) 'Central intake to improve access to physiotherapy for children with complex needs: A mixed methods case report', *BMC Health Services Research*. BioMed Central Ltd., 16(1). doi: 10.1186/s12913-016-1700-3.
87. Xu, Y. *et al.* (2017) 'Predicting Changes in Pediatric Medical Complexity using Large Longitudinal Health Records.', *AMIA ... Annual Symposium proceedings. AMIA Symposium*, 2017, pp. 1838–1847.
